# A Case of Ovarian Teratoma–Associated Anti‐NMDA Receptor Encephalitis in the Presence of Group A Streptococcus Infection

**DOI:** 10.1155/crog/1359794

**Published:** 2026-06-23

**Authors:** Anna Blach, Shannon Kiss, Alexandra Fontaine, Robert Graebe

**Affiliations:** ^1^ Department of Obstetrics and Gynecology, Monmouth Medical Center, Long Branch, New Jersey, USA, rwjbh.org; ^2^ Department of Graduate Medical Education, Rowan University School of Osteopathic Medicine, Stratford, New Jersey, USA, rowan.edu

**Keywords:** anti-NMDAR encephalitis, Group A Streptococcus, gynecology, neurology, ovarian teratoma, psychiatry

## Abstract

**Background and Aims:**

Anti‐NMDAR encephalitis is a severe autoimmune encephalitis that is frequently associated with ovarian teratomas, whereas infectious triggers remain incompletely understood. We report a case of ovarian teratoma–associated anti‐NMDAR encephalitis in a young woman with concurrent Group A Streptococcus infection presenting with prominent psychiatric symptoms and subsequent neurologic decline.

**Methods:**

We describe the clinical course, diagnostic evaluation, and multidisciplinary management of a 25‐year‐old African American woman with acute‐onset neuropsychiatric symptoms, confirmed anti‐NMDAR encephalitis, bilateral ovarian teratomas, and concurrent Group A Streptococcus infection. Verbal informed consent was obtained from the patient for publication of this case report and any accompanying images.

**Results:**

The patient initially presented with acute psychiatric symptoms and was treated for suspected schizoaffective disorder. Further evaluation following clinical deterioration revealed anti‐NMDAR antibodies in the cerebrospinal fluid and bilateral ovarian teratomas on imaging. She was treated with IVIG, high‐dose corticosteroids, bilateral ovarian cystectomy, and plasma exchange, with subsequent clinical improvement and discharge after prolonged hospitalization.

**Conclusion:**

This case underscores the importance of considering anti‐NMDAR encephalitis in young women presenting with acute psychiatric symptoms and neurologic decline. It also highlights the importance of pelvic imaging for ovarian teratoma and careful infectious evaluation, including for streptococcal infection when clinically indicated. The observed association between Group A Streptococcus infection preceding symptom onset suggests a possible infectious trigger leading to an immunologic response and subsequent anti‐NMDAR encephalitis; however, there is limited evidence of a causal relationship in the literature.

## 1. Background

The discovery of psychiatric manifestations in the context of encephalitis was first described in four young women with ovarian teratomas by Vitaliani et al. in 2005 [1]. These acute psychiatric symptoms included seizures, decreased levels of consciousness, hypoventilation, and memory deficits that correlated with inflammatory abnormalities in the cerebrospinal fluid (CSF) [1]. Following the early work of Vitaliani et al. [1] who linked psychiatric symptoms with ovarian teratomas, Dalmau et al. [2] further characterized anti‐NMDAR encephalitis, providing laboratory evidence of antibodies against NMDA receptors, particularly in women with teratomas.

**Figure 1:**
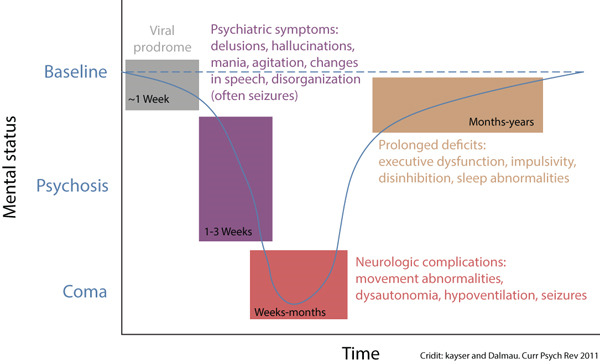
Anti‐NMDAR encephalitis clinical mental status change progression and prognosis [3, 4].

The manifestations of symptoms typically begin with prodromal symptoms, followed by acute psychiatric symptoms that progress to severe neurologic symptoms such as autonomic instability, seizures, and respiratory depression (Figure 1) [5]. According to Titulaer et al. [3], approximately 80% of patients return to a near‐baseline level of functioning after tumor removal, when present, and early aggressive immunosuppression. New data suggest that relapse is less common than previously hypothesized, with an approximate 10%–15% chance of recurrence within a 2‐year period when patients are treated aggressively [3, 4].

Ovarian teratomas are thought to provoke an autoimmune response against the NMDA receptor in the brain, as the teratoma tissue expresses similar antigens, particularly the NR1 protein, which triggers the production of autoantibodies that bind to and disrupt NMDA receptor function in the central nervous system (CNS). Ovarian teratomas are the most common ovarian tumor in women; however, only a very small percentage of patients who have them develop anti‐NMDAR encephalitis, suggesting that additional factors could be involved. Given that prodromal symptoms occur in 75% of these cases, infection has been proposed as a possible inciting factor. Although HSV is the most well‐established infection associated with anti‐NMDAR encephalitis, there have also been reports implicating other pathogens such as *Mycoplasma*, influenza, and rhinovirus, with some evidence suggesting possible associations with Group A Streptococcus (GAS) [6–9].

In this report, we present a case of a young patient with anti‐NMDAR encephalitis and bilateral ovarian teratomas, with evidence of Group A Streptococcal infection preceding her neuronal dysfunction.

## 2. Case Presentation

The patient, a 25‐year‐old African American female with a history of chronic hypertension, was escorted to the emergency department by her mom with acute psychiatric symptoms. Her clinical course progressed rapidly from psychiatric symptoms to recurrent loss of consciousness, autonomic instability, and confirmed anti‐NMDAR encephalitis associated with bilateral ovarian teratomas. Her mother reported a 2‐week history of increasingly unusual behavior, including decreased sleep, irritability, and unprovoked screaming, which acutely worsened over the 5 days prior to presentation. Notably, the patient had complained of a sore throat and decreased hearing in her right ear about a week prior to symptom onset. During her initial evaluation upon presentation to the hospital, the patient was erratic—singing and screaming as well as conversing with a person not there. She became combative towards the staff with threatening gestures. She was not redirectable and so was medicated with Haldol and Ativan. Her initial vital signs were in normal range: blood pressure of 122/84, heart rate of 88, respiratory rate of 18, and temperature of 98.9 F. Initial bloodwork was also within normal limits as follows: Hgb of 13.5, WBC of 10.30, platelets of 271, creatinine of 0.80, glucose of 103, ALT/AST of 23/34, and electrolytes within the normal range. A urine toxicology screen and pregnancy test were both negative. A head CT scan was unremarkable. She was admitted to the psychiatry unit under an involuntary hold for suspected schizoaffective disorder (bipolar type) and treated with Depakote, Risperdal, and Ativan. On admission, the patient was found to have a positive PCR test for Group A Streptococcus, and she was treated with a 7‐day course of amoxicillin.

On hospital day 5, the patient experienced a sudden loss of consciousness and sustained head trauma. Her diagnosis of schizoaffective disorder was revisited after the lack of improvement, and new neurological signs prompted further investigation into an organic cause. A second CT scan was again unremarkable. Lumbar puncture revealed rare lymphoid cells; however, the cell yield was insufficient for full interpretation. HSV‐1 and HSV‐2 DNA were negative. A full infectious workup was negative (aside from the Group A Streptococcus noted on admission). An EEG showed 7–8 Hz theta and alpha posterior dominant rhythm, normal anterior to posterior gradient, with superimposed 15–20 Hz beta activity, likely secondary to medication, and no lateralizing or epileptiform findings noted. A brain MRI showed no acute intracranial abnormalities.

On hospital day 9, a second lumbar puncture was performed which now revealed 1:32 anti‐NMDA receptor antibodies. Additional lumbar puncture findings were as follows: glucose of 73, protein of 24.2, clear, 4 WBC, 1 RBC, 86 lymphocytes, 14 monocytes, and lactate CSF of 2.36, no oligoclonal bands noted, CSF VRDL nonreactive, and neuron‐specific enolase 6.3 (negative). CSF bacterial and viral cultures showed no growth. She was diagnosed with anti‐NMDAR encephalitis and started on IVIG (0.4 g/kg daily). After 5 days of IVIG therapy, there was no clinical improvement, and she was subsequently started on methylprednisolone 1000 mg IV q24h. A CT scan of the abdomen and pelvis demonstrated several bulky, fat‐containing mixed cystic lesions arising from both ovaries/adnexa, most compatible with teratomas, favoring mature cystic ovarian teratomas given their smooth borders and lack of locally aggressive features.

**Figure 2:**
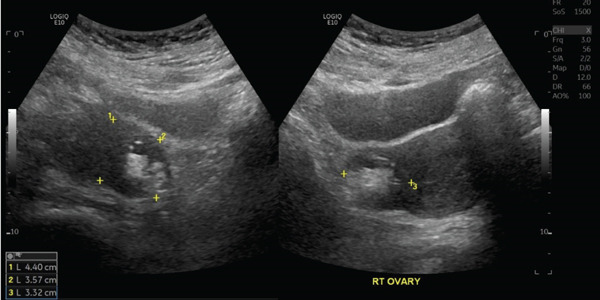
Abdominal pelvic US showed right adnexal heterogeneous mixed cystic and solid, fat‐containing lesion.

**Figure 3:**
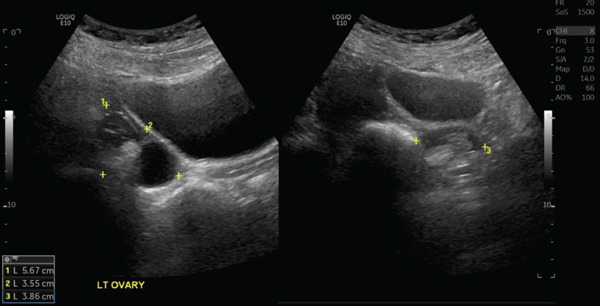
Abdominal pelvic US showed left adnexal heterogeneous mixed cystic and solid, fat‐containing lesion.

The gynecology service was consulted for possible surgical management of suspected ovarian teratomas. An abdominal pelvic US showed right adnexal 3.1 × 2.5 × 2.7 cm heterogenous mixed cystic and solid, fat‐containing lesion (Figure 2) and left adnexal 4.6 × 3.4 × 3.8 cm heterogenous mixed cystic and solid, fat‐containing lesion (Figure 3). As the patient was unable to provide consent, a discussion was held with her family to recommend the removal of bilateral teratomas via bilateral ovarian cystectomy, and possibly bilateral oophorectomy if indicated. Given the risk of surgical menopause and sterilization, the patient′s sister wished to discuss with her family before proceeding with surgery.

In the days that followed, the patient was noted to be febrile and tachycardic. Imaging studies showed lung atelectasis with basilar infiltrate. Serial electrocardiograms performed showed sinus tachycardia with intermittent sinus pauses (up to 7 s), and a TTE showed mild concentric hypertrophy. She was treated empirically with Zosyn, vancomycin, and ceftriaxone; yet again, infectious workup was negative.

On hospital day 17, the patient′s family notified the team that they agreed to move forward with surgery. She received medical and cardiac clearance. An exploratory laparotomy and bilateral ovarian cystectomy via Cherney incision was performed. During the surgery, the left ovary had an approximately 4‐cm ovarian cyst with fat globules; the right ovary had one approximately 1.5‐cm cyst and 1‐cm fluid‐filled cysts. The cysts did not grossly appear to be dermoids; however, all three were removed intact. The final pathology report showed that all three cysts removed were benign, mature cystic teratomas with keratin, hair, and mucus noted. The left dermoid was 3 × 2.5 × 1.5 cm (5 g) and the right dermoids were 1.5 × 1 × 1 cm (1 g) and 2.2 × 1.7 × 0.5 cm (2 g).

Following the cyst removal, the patient remained with minimal communication; however, improvements were noted in that she did not require the use of 4‐point restraints and was less agitated, no longer requiring risperidone QHS. Plasma exchange for a full course of 7 treatments was initiated on postoperative day 4. The patient continued to show signs of clinical improvement and was extubated on postoperative day 21 and required significant physical rehabilitation. She was ultimately discharged home after 51 days in the hospital with outpatient follow‐up with neurology, psychiatry, OBGYN, and PCP.

## 3. Discussion

Encephalitis is an inflammatory condition of the brain characterized by neuropsychiatric symptoms often followed by rapid neurological deterioration. The causes of encephalitis are myriad, with both infectious and autoimmune etiologies [2]. Initially described in 2007 with five women who had encephalitis with mature ovarian teratomas and laboratory evidence of the target NMDA receptor antigen, anti‐NMDAR encephalitis is now the most common type of autoimmune encephalitis. Since 2005, there have been over 800 cases reported in the literature [10–18].

This report describes a rare constellation of findings in an adult woman with bilateral mature cystic ovarian teratomas, concurrent laboratory‐confirmed Group A Streptococcus infection, anti‐NMDA receptor antibodies, and substantial clinical improvement following combined immunotherapy and surgery. To our knowledge, such a constellation has rarely been reported. This case does parallel prior reports of teratoma‐associated anti‐NMDAR encephalitis, including the case described by Koenig et al. [10], and highlights the importance of prompt diagnosis and multidisciplinary management.

NMDARs (N‐methyl‐D‐aspartate‐receptor) are ligand‐gated ion channels distributed widely in the brain that are vital to synaptic function and neuropsychiatric health. NMDA receptors play a key role in excitatory neurotransmission and synaptic plasticity. In NMDAR‐encephalitis, autoantibodies target the NR1 subunit of these receptors, leading to receptor internalization and neuronal dysfunction.

Ovarian teratomas are present in an estimated 30%–60% of cases of anti‐NMDAR encephalitis in reproductive‐aged females, making ovarian teratoma the most common known cause [18]. In these cases, immune recognition of the NR1 protein within the teratoma leads to germinal center formation, B‐cell activation, and antibody production [19] with subsequent activation of NMDA receptors in the brain and clinical sequelae. Ovarian teratomas are the most common tumors of the ovary, yet only a small subset of patients with these tumors develop this condition. In 2022, the Journal of Clinical Neuroscience identified a cohort of 24,270 patients admitted for an ovarian dermoid tumor. Of this sample group, only 50 (0.21%) were diagnosed with anti‐NMDAR encephalitis [20]. The low incidence suggests that there could potentially be other factors or triggers present in patients with teratomas that initiate anti‐NMDAR encephalitis.

A prodromal phase, including symptoms such as fever, headache, and upper respiratory infection, is seen in many cases and has led to the hypothesis that infection may act as a trigger [5, 19, 21, 22]. Although HSV is the most commonly linked pathogen, infections such as mycoplasma, influenza, rhinovirus, and chikungunya have also been implicated in some cases, although the exact mechanisms remain unclear [6–8, 21, 22] Proposed mechanisms include molecular mimicry, the unmasking of cryptic epitopes in the setting of tissue damage, the breaking of immune tolerance, and breaking of the blood–brain barrier [21, 22]. A study in 2024 analyzed teratomas of encephalitic dermoid tumors compared with nonencephalitic tumors. The results of that study showed that there were no significant differences in the distribution of NR‐1+ cells within the ovarian teratomas and whole‐exome sequencing showed no significant differences in the canonical mutations of the two groups, suggesting no genetic differences. Additionally, the encephalitic tumors showed higher germinal center formation compared with the nonencephalitic control teratomas [9]. Taken together, these results support the hypothesis that some type of infectious process triggers the autoimmune response to the teratoma.

In the case we present, the patient complained of a sore throat and unilateral hearing loss approximately 1 week prior to the onset of her symptoms. Then on admission, testing was positive for Group A strep. We hypothesize that a preceding streptococcal infection was perhaps the trigger for the anti‐NMDAR encephalitis. Although no direct adult cases linking Group A Streptococcus to anti‐NMDAR encephalitis have been reported, pediatric data suggest an association between recent streptococcal infection and anti‐NMDAR encephalitis [7]. Accordingly, the temporal association observed in our patient should be interpreted as hypothesis‐generating rather than causal.

This hypothesis is biologically plausible given the well‐known cross‐reactive antigens of Group A Streptococcus. The bacterium has known cross‐reactive antigens, including M‐protein and N‐acetyl‐*β*‐D‐glucosamine, that mimic host molecules and induce an immune response against host tissues. This pathobiology is well understood and explains acute rheumatic fever, rheumatic heart disease, and Sydenham chorea. Sydenham chorea, specifically, is a sequela of rheumatic fever in which monoclonal antibodies specific to both human lysoganglioside and the carbohydrate group on GAS—target the surface of neuronal cells. Additionally, in PANDAS (Pediatric Autoimmune Neuropsychiatric Disorder Associated with Streptococcal Infection) there are similar antineuronal autoantibodies and subsequent neuronal dysfunction [23]. As previously mentioned, the brain is an immune‐privileged organ and protected by the blood–brain barrier. Studies of mice have demonstrated that intranasal group A streptococcal infection leads to activated Th17 cells that traverse the blood–brain barrier and allow influx of immunoglobulins [23].

This report has several important limitations. It is a single‐case report, and no definitive causal relationship can be established between concurrent Group A Streptococcus infection and the onset of anti‐NMDAR encephalitis. In addition, because ovarian teratomas themselves are a well‐established trigger for anti‐NMDAR encephalitis, the relative contribution of infection in this case cannot be determined. Nonetheless, the temporal sequence and biologic plausibility support further investigation of infectious triggers in future studies.

Additionally, we present a success story of a patient who was treated and had a meaningful neurologic recovery. Our treatment course included surgical removal of the teratoma, IVIG, high‐dose steroids, and plasma exchange. Because these therapies were administered sequentially, the relative contribution of each cannot be isolated; however, our patient′s improvement following combined immunotherapy and surgical removal of the teratomas underscores the importance of prompt multidisciplinary intervention in cases of anti‐NMDAR encephalitis with ovarian teratomas.

Further, in cases of neuronal dysfunction of unknown origin, anti‐NMDAR encephalitis should be included in the differential. In diagnosed cases, imaging should be performed on all women to evaluate for the presence of teratomas. Given how small the teratomas may be that cause disease, careful multidisciplinary consideration of additional imaging or surgical evaluation may be warranted when clinical suspicion remains high despite equivocal imaging findings.

## Funding

No funding was received for this manuscript.

## Disclosure

All authors have read and approved the final version of the manuscript. Robert Graebe, M.D. had full access to all of the data in this study and takes complete responsibility for the integrity of the data and the accuracy of the data analysis. Robert Graebe, M.D. affirms that this manuscript is an honest, accurate, and transparent account of the study being reported; that no important aspects of the study have been omitted; and that any discrepancies from the study as planned have been explained.

## Conflicts of Interest

The authors declare no conflicts of interest.

## Data Availability

The authors confirm that the data supporting the findings of this study are available within the article and its supporting information.
